# Endothelial Cell-Derived Extracellular Vesicles Allow to Differentiate Between Various Endotypes of INOCA: A Multicentre, Prospective, Cohort Study

**DOI:** 10.1007/s12265-024-10575-x

**Published:** 2024-12-05

**Authors:** Aleksandra Gąsecka, Piotr Szolc, Edwin van der Pol, Łukasz Niewiara, Bartłomiej Guzik, Paweł Kleczyński, Mariusz Tomaniak, Emilia Figura, Mateusz Zaremba, Marcin Grabowski, Janusz Kochman, Jacek Legutko, Łukasz Kołtowski

**Affiliations:** 1 1st Chair and Department of Cardiology, Medical University of Warsaw, Banacha 1a, 02-097 Warsaw, Poland; 2 Amsterdam Vesicle Center, Amsterdam UMC, University of Amsterdam, Amsterdam, The Netherlands; 3 Department of Emergency Medicine, Faculty of Health Sciences, Jagiellonian University Medical College, Kraków, Poland; 4 Clinical Department of Interventional Cardiology, Saint John Paul II Hospital, Krakow, Poland; 5 Amsterdam UMCBiomedical Engineering & PhysicsLaboratory of Experimental Clinical Chemistry, University of Amsterdam, Amsterdam Cardiovascular Sciences, Atherosclerosis & Ischemic Syndromes, Meibergdreef 9, Amsterdam, The Netherlands; 6 Faculty of Medicine, Institute of Cardiology, Department of Interventional Cardiology, Jagiellonian University Medical College, Krakow, Poland

**Keywords:** Coronary microcirculatory disease, INOCA, Vasospastic angina

## Abstract

Ischemia and non-obstructive coronary artery disease (INOCA) might be due to coronary microvascular dysfunction (CMD), vasospastic angina (VSA) or both. We compared plasma concentration of various extracellular vesicles (EVs) in patients with different INOCA endotypes. Patients were divided into those with INOCA (CMD, VSA, mixed CMD + VSA) and non-anginal chest pain. Plasma concentrations of EVs were measured using flow cytometry. Out of 96 patients included, 34 had CMD (35%), 15 VSA (16%), 24 mixed endotype (25%) and 23 non-anginal chest pain (24%). Patients with INOCA had lower ratio of endothelial EVs (CD144 +) to total EVs, compared to patients with non-anginal pain (*p* = 0.027). Patients with mixed endotype had lower ratio of endothelial EVs (CD144 +) to total EVs, compared to CMD (*p* = 0.008), VSA (*p* = 0.014) and non-anginal pain (*p* < 0.001). Decreased ratio of endothelial EVs (CD144 +) to total EVs might serve as a "circulating footprint" of the mixed INOCA endotype.

**Graphical Abstract:**

CCS – chronic coronary syndrome, CD – cluster of differentiation, CMD – coronary microvascular dysfunction, CFR – coronary flow reserve, EVs – extracellular vesicles, FFR – fractional flow reserve, INOCA - IMR – index of microvascular resistance, VSA – vasospastic angina

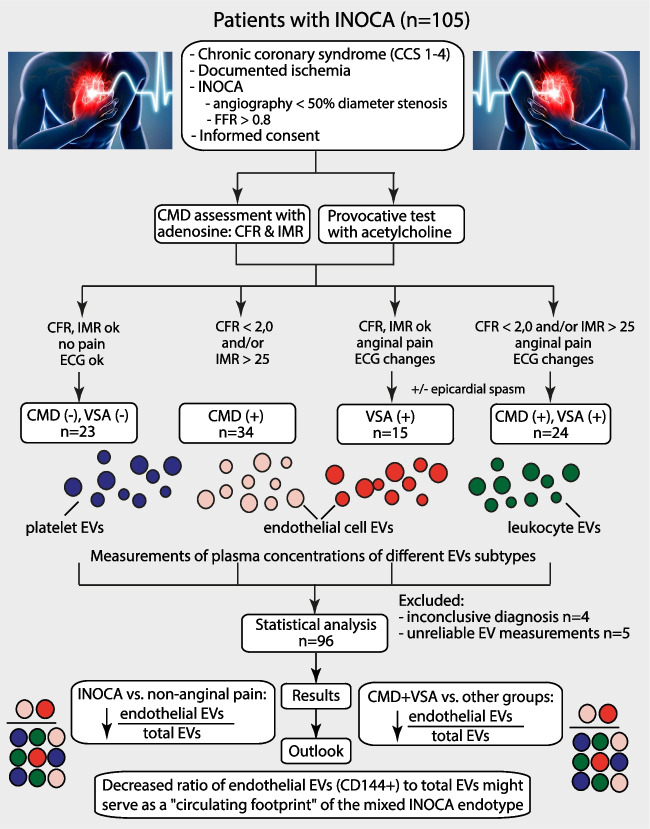

**Supplementary Information:**

The online version contains supplementary material available at 10.1007/s12265-024-10575-x.

## Introduction

Half of patients undergoing coronary angiography have ischemia and non-obstructive coronary artery disease non-obstructive coronary artery disease despite clinical symptoms and evidence of ischemia (INOCA) [1]. Recently, we published data from the MOSAIC-COR registry, showing that roughly 10% of INOCA patients have isolated coronary microvascular dysfunction (CMD), 40% of them present with epicardial or microvascular vasospastic angina (VSA), 40% with mixed CMD and VSA, and only 10% with non-cardiac chest pain [2]. The persistent anginal symptoms of these patients significantly worsens their quality of life and poses a financial burden to the healthcare system [3].

For diagnosis, patients with INOCA undergo repeated computed tomography angiography or coronary angiography. The presence of objectively confirmed CMD or VSA increases both the frequency of adverse cardiovascular events and mortality [4, 5]. To mitigate this risk, β-blockers, angiotensin-converting enzyme (ACE) inhibitors and statins are recommended in CMD patients, whereas calcium antagonists and long-acting nitrates should be started in VSA patients, along with lifestyle changes [6, 7]. Therefore, identification of the specific INOCA endotype is a key element to guide pharmacotherapy, provide prognostic information to patients and physicians, improve outcomes and decrease healthcare costs.

Currently, the diagnostic algorithm in patients with INOCA requires coronary function testing. After excluding a significant epicardial stenosis in coronary angiography, invasive measurement of coronary flow reserve (CFR) and index of microcirculatory resistance (IMR) during intravenous infusion of vasodilator (e.g. adenosine) is required [6]. According to CorMicA definition, CMD is defined as CFR < 2.0 or IMR ≥ 25 units [1, 8]. The diagnosis of VSA, in turn, requires a provocative test using acetylcholine (ACh). VSA is confirmed in patients with anginal symptoms and ST segment deviations of ≥ 1 mm in a 12-lead ECG, accompanied by a 90% spasm of an epicardial artery (epicardial VSA) or without such spasm (microvascular spasm) [1, 8]. However, guidewire-based CMD diagnosis is an imperfect method both due to guidewire failure including kinking, communication failure or shaft fracture, or patient-related adverse events including retained guidewire tip and coronary artery dissection [9]. Similarly, ACh provocative test is associated with an inherent risk of complications, including diffuse epicardial spasm in about 20% of patients, posing a risk of peri-procedural myocardial infarction in elective patients [10]. In another 20% of patients, the provocative test is inconclusive due to the absence of changes in the 12-lead ECG, which does not allow to assess certain myocardial areas, especially within the right ventricle and posterior wall. Hence, novel diagnostic methods to establish the endotype of INOCA are urgently required.

**Fig. 1:**
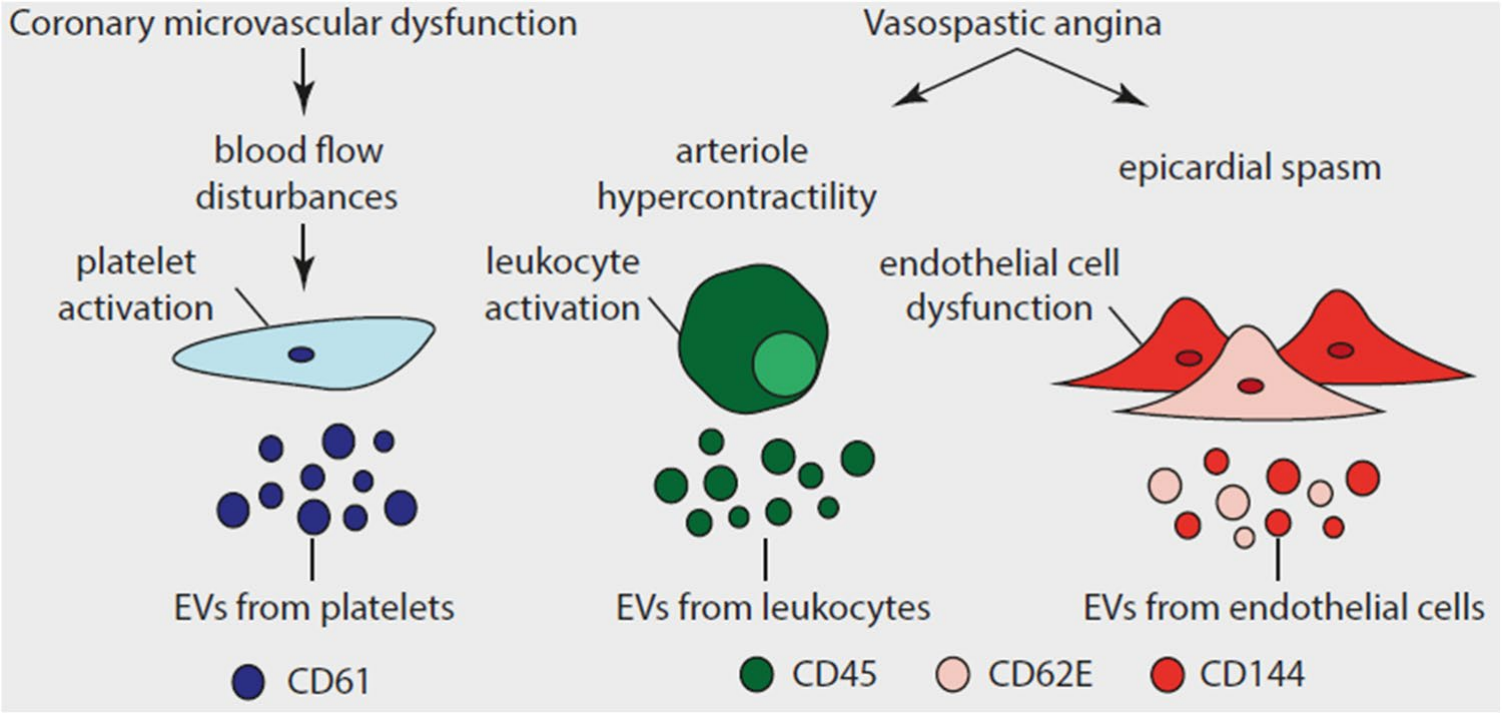
Research hypothesis. CD – cluster of differentiation

Regarding pathophysiology, CMD results mostly from impaired vasodilation due to vascular smooth muscle cells (VSMCs) damage, whereas VSA is caused by increased vasoconstriction due to endothelium damage [11]. Damaged VSMCs and endothelial cells, but also other blood cells such as platelets and leukocytes release extracellular vesicles (EVs). EVs are nanoparticles which contain cytoplasmic material surrounded by a lipid bilayer and expose antigens derived from the parent cells, allowing to identify their origin. EVs participate in key processes underlying atherosclerosis, including endothelial dysfunction and VSMC proliferation [12]. There are several subpopulations of EVs from activated endothelial cells, including EVs exposing VE-cadherin (CD144) and E-selectin (CD62E) [13]. EVs from platelets, in turn, can be identified based on the exposure of glycoprotein IIIa (CD61), a receptor for fibrinogen responsible for cross-linking of adjacent platelets during thrombus formation [12]. Plasma concentrations of endothelial cell- and leukocyte-derived EVs reflect the degree of endothelial damage in atherosclerosis [13]. Coronary artery spasm, in turn, is associated with impaired platelet nitric oxide signalling and an increase in platelet-derived EV concentrations [14]. Considering the different endotypes of INOCA with divergent pathophysiology, we hypothesized that EVs might serve as a "circulating footprint" indicating the specific INOCA endotype (Fig. 1). The objective of this study was to compare the plasma concentration of various EV subtypes between (i) patients with and without INOCA, and (ii) patients with different INOCA endotypes and non-anginal chest pain.

## Methods

### Study Design

This was a prospective study conducted at two high-volume academic centers (1^st^ Chair and Department of Cardiology, Medical University of Warsaw, Poland and Department of Interventional Cardiology, Institute of Cardiology, Jagiellonian University Medical College, Krakow, Poland) in collaboration with Amsterdam Vesicle Center, Amsterdam University Medical Centers, the Netherlands. The study protocol, designed in compliance with the Declaration of Helsinki, was approved by the Ethics Committee of Medical University of Warsaw (approval number: KB/105/2021) and the Ethics Committee of Medical Chamber in Krakow (approval number: 304/KBL/OIL/2019).

### Selection of Participants

The study population included patients with chronic coronary syndrome (CCS 1–3), objectively documented myocardial ischemia (i.e. ischemic changes on ECG during chest pain episode; reversible abnormalities in stress myocardial perfusion; reversible abnormalities in contractility on stress echocardiography), absence of significant obstructive coronary artery disease (CAD) (< 50% diameter stenosis in angiography or fractional flow reserve [FFR] > 0.80) and informed consent to participate in the study. Exclusion criteria were (i) indications for coronary angiography other than CAD (i.e. valvular disease, post-cardiac arrest state, left ventricular systolic dysfunction with ejection fraction [EF] < 30%), (ii) detection of at least one significant stenosis in at least one of the major epicardial arteries with a diameter > 2.5 mm (> 50% diameter stenosis and/or FFR < 0.8), (iii) tortuosity or calcifications within the coronary arteries preventing FFR guidewire insertion, (iv) history of PCI with stent implantation, (v) prolonged QTc interval on ECG, (vi) chronic kidney disease (estimated glomerular filtration rate [eGFR] < 30 ml/min/1.73 m^2^), (vii) severe chronic obstructive pulmonary disease (GOLD 4), (viii) allergy to iodinated contrast agents, (ix) pregnancy and breast-feeding, and (x) lack of patient informed consent to participate in the study.

### Coronary Function Testing

Coronary angiography was performed in line with the standard of care at the participating hospitals, and coronary function tests were performed as adjunctive procedures. Coronary microvascular function was assessed using CoroFlow™ Software (Coroventis, Uppsala, Sweden) and PressureWire X (Abbott, Illinois, US). A pressure–temperature sensor was inserted into the left anterior descending artery (LAD) territory. In case of severe LAD tortuosity, another epicardial artery which allowed for FFR wire insertion was chosen. Following intracoronary administration of 200 µg of nitroglycerin, FFR, CFR and IMR were measured in real-time at rest and during hyperaemia using thermodilution method. Hyperaemia was achieved by an intravenous infusion of adenosine (140 µg/kg/min). Thermodilution was performed by repeated intracoronary injections of 3 ml of saline. Subsequently, ACh was administered intracoronary in incremental doses according to standardized protocol during continuous 12-leasd ECG monitoring. In case of anginal pain and ischaemic ECG changes allowing to diagnose VSA with the smaller ACh dose, the higher doses were not administered. Following the provocation test, nitroglycerin was administered intracoronary until the resolution of the spasm, anginal pain and/or ECG changes. Based on the coronary function testing results, patients were divided as proposed in the CorMicA trial: patients with INOCA (CMD, VSA or mixed endotype) and patients without INOCA (non-anginal chest pain, i.e. no CMD or VSA).

### Sample Collection and Handling

Venous blood was collected from all patients once, prior to coronary angiography. Blood was collected from antecubital vein into 7.5 mL 0.109 mol/L ethylenediaminetetraacetic acid (EDTA) plastic tubes (S-Monovette, Sarstedt) according to the guidelines to study EVs and processed by trained professionals (P.S., E.F., M.Z) [12]. Within 1 h from blood collection, platelet-depleted plasma was prepared by double centrifugation. The centrifugation parameters were: 2500 g, 15 min, 20 °C, acceleration speed 1, no brake. The first centrifugation step was done with 7.5 mL whole blood collection tubes. Supernatant was collected 10 mm above the buffy coat. The second centrifugation step was done with 3.5 mL plasma in 15 mL polypropylene centrifuge tubes (Greiner Bio-One B.V). Supernatant (platelet-depleted plasma) was collected 5 mm above the buffy coat, transferred into 5 mL polypropylene centrifuge tubes (Greiner Bio-One B.V.), mixed by pipetting, transferred to 1.5 mL low-protein binding Eppendorfs (Thermo Fisher Scientific), and stored at − 80 °C until analysis. Prior to analysis, samples were thawed for 1 min in a water bath (37 °C) to avoid cryoprecipitation.

### Laboratory Assays

Laboratory assays were conducted by EVcount (Amsterdam, The Netherlands), a startup company of the Amsterdam University Medical Centers specialized in EV concentration measurements. To determine the concentration of EV subtypes in platelet-depleted plasma, flow cytometry (A60-Micro, Apogee Flow Systems) was used. The reported concentrations describe the number of particles (1) that exceeded the side scattering threshold, corresponding to a side scattering cross section of 10 nm^2^, (2) with a diameter > 200 nm as determined by the flow cytometry scatter ratio (Flow-SR) [15], (3) having a refractive index < 1.42 to omit false positively labeled chylomicrons [16] and (4) that are positive at the fluorescence detectors corresponding to the used labels, per mL of platelet-depleted plasma. We defined the following EV subtypes: EVs derived from endothelial cells (CD144 + and CD62E +), leucocytes (CD45 +) and platelets (CD61 +). In addition, we defined the total concentration of EVs as the events fulfilling aforementioned criteria 1 to 3. Results are shown as ratio of the concentrations of EV subtypes to total EV concentrations.

To improve the reproducibility of EV flow cytometry experiments, EVcount (i) applied the framework for standardized reporting of EV flow cytometry experiments (MIFlowCyt-EV) [17] (ii) calibrated all detectors, (iii) determined the EV diameter and refractive index by Flow-SR [15] and (iv) applied custom-built software to fully automate data calibration and processing [18]. All relevant details about assay controls, instrument calibration, data acquisition, and EV characterization are included in the Supplementary File.

### Endpoints

The primary endpoint was the difference in concentrations of EV subtypes in patients with INOCA and non-anginal chest pain. The secondary endpoint was the difference in concentrations of EV subtypes depending on the INOCA endotype (CMD vs. VSA vs. mixed endotype vs. non-anginal chest pain).

### Statistical Analysis

Currently, there is no data regarding the differences in EV concentrations between patients with and without INOCA or with different INOCA endotypes. Hence, the sample size was calculated based on the differences in platelet, leukocyte and endothelial EV concentrations between patients with CAD and healthy individuals, as demonstrated in the recent meta-analysis [19]. Patients with CAD had nominally twofold higher concentrations of the investigated EV subtypes compared to healthy controls. Concurrently, we assumed that the (i) mean difference in EVs concentrations between patients with and without INOCA = 1, (ii) standard deviation (SD) ± 1.0, and (iii) nominal test power = 0.9. Based on these assumptions, each group should include at least 23 patients (a total of 46 patients). Assuming INOCA rate of 50%, at least 92 patients should be enrolled in the study [1].

Statistical analyses were conducted using IBM SPSS Statistics, version 27.0 (IBM, New York, USA). Categorical variables were presented as number and percent and compared using χ2 test. Shapiro–Wilk test was used to assess normal distribution of continuous variables. Continuous variables were presented as mean with standard deviation (SD) or median with interquartile range (IQR). Differences in EV concentrations in patients with and without INOCA were compared using unpaired t-test or U-Mann Whitney, depending on data distribution. Differences in EV concentrations in patients with different INOCA endotypes were compared using Kruskal–Wallis test with Bonferroni correction for multiple comparisons. A chi-square test was used to compare categorical variables. Spearman correlation coefficient was used to evaluate correlations between EVs and echocardiographic parameters and B-type natriuretic peptide (BNP).

The diagnostic value of EVs for INOCA and the cut-offs were calculated using a receiver operating characteristic (ROC) curve. Logistic regression model incorporating EVs and clinical characteristics which differed between patients with and without INOCA were used to determine independent variables associated with the diagnosis of INOCA. The results of multivariable regression analysis are reported as odds ratio (OR) and 95% confidence interval (CI). A two-sided p-value below 0.05 was considered significant.

## Results

The study design, flow chart and study results are shown in the Graphical Abstract. Between December 2021 and March 2023, 105 patients were enrolled and 96 patients were included in the analysis: 34 with CMD (35%), 15 with VSA (16%), 24 with mixed endotype (25%) and 23 with non-anginal chest pain (24%). The clinical characteristics of the patients are presented in Table 1. Regarding baseline characteristics, there were significant differences regarding body mass index (BMI) and New York Heart Association (NYHA) class (*p* = 0.008,* p* = 0.019, respectively), i.e. patients with VSA having the lowest BMI, and patients with VSA + CMD presenting with the highest NYHA class, compared to other groups. As expected, patients with CMD and VSA + CMD had lower CFR and highest IMR, compared to other groups (*p* < 0.001, *p* = 0.002, respectively). In terms of pharmacotherapy, patients with VSA and VSA + CMD received beta blockers less often and non-dihydropyridine calcium channel blockers more often than patients with CMD only or with non-anginal pain (*p* = 0.005, *p* = 0.001, respectively). There were no other differences between the groups.

**Table 1:** Baseline characteristics. Number of patients: 96. Continuous data are presented as mean (standard deviation) or median (interquartile range) and compared using Kruskal–Wallis test. Categorical variables are presented as number (%) and compared using Chi-square test. Significant p-values are bold

Characteristic	CMD (*n* = 34)	VSA (*n* = 15)	CMD + VSA (*n* = 24)	Non-anginal pain (*n* = 23)	*p*-value
Age, years	66 (9)	58 (10)	62 (11)	65 (11)	0.064
Female	27 (79%)	9 (60%)	13 (54%)	15 (65%)	0.213
BMI, kg/m^2^	30 (4)	26 (4)	31 (5)	31 (4)	**0.008**
Hypertension	30 (88%)	10 (67%)	21 (88%)	22 (96%)	0.112
Dyslipidemia	31 (91%)	13 (87%)	19 (79%)	20 (87%)	0.606
Smoking					0.248
Current	5 (15%)	3 (20%)	2 (8%)	5 (22%)	
Past	4 (12%)	4 (27%)	8 (33%)	2 (9%)	
Diabetes	11 (32%)	4 (27%)	5 (21%)	5 (22%)	0.764
Prior angiography	11 (32%)	6 (40%)	7 (29%)	6 (26%)	0.834
Prior ACS	2 (6%)	3 (20%)	5 (21%)	3 (13%)	0.350
Prior stroke	2 (6%)	1 (7%)	2 (8%)	0 (0%)	0.614
**Symptoms**					
CCS class					0.107
0	3 (9%)	4 (27%)	6 (25%)	7 (30%)	
I	5 (15%)	0 (0%)	4 (17%)	0 (0%)	
II	19 (56%)	10 (67%)	10 (42%)	13 (57%)	
III	5 (15%)	0 (0%)	4 (17%)	2 (9%)	
NYHA class					**0.019**
0	23 (68%)	13 (87%)	16 (67%)	11 (48%)	
I	0 (0%)	0 (0%)	2 (8%)	0 (0%)	
II	8 (24%)	2 (13%)	1 (4%)	8 (35%)	
III	1 (3%)	0 (0%)	4 (17%)	2 (9%)	
Treadmill test	11 (32%)	9 (60%)	12 (50%)	5 (22%)	0.532
SPECT	5 (15%)	3 (20%)	7 (29%)	5 (22%)	0.773
MR	0 (0%)	0 (0%)	0 (0%)	1 (4%)	0.396
**Laboratory parameters**					
Hemoglobin, g/dl	13.4 (1.3)	14.2 (1.5)	14.2 (1.5)	13.8 (1.4)	0.145
GFR, ml/min	74 (20)	85 (13)	78 (19)	77 (19)	0.393
BNP, pg/ml	271 (271)	161 (154)	206 (262)	284 (277)	0.402
Total cholesterol, mg/dl	174 (47)	155 (33)	184 (37)	171 (39)	0.201
LDL-C, mg/dl	102(49)	83(22)	106(37)	102(35)	0.408
**Echocardiography**					
LVEF	59 (6)	61 (6)	60 (6)	59 (6)	0.910
LVEDD	45 (6)	48 (5)	48 (5)	49 (6)	0.154
IVS	10 (1)	10 (2)	10 (3)	11 (4)	0.824
PWT	10 (1)	10 (2)	10 (1)	10 (2)	0.831
**Procedural details**					
Contrast volume, ml	153 (38)	136 (35)	175 (61)	185 (68)	0.112
Radiation dose, mGy	464 (444)	574 (494)	564 (410)	641 (409)	0.071
Slow flow	4 (12%)	4 (27%)	2 (8%)	2 (9%)	0.400
LVEDP	13.1 (7.2)	8.5 (4.4)	12.3 (7.2)	13.7(5.3)	0.354
LAD examined	33 (97%)	15 (100%)	24 (100%)	23(100%)	1.000
RFR	0.93 (0.02)	0.93(0.03)	0.92 (0.02)	0.93 (0.02)	0.157
FFR	0.90 (0.05)	0.91 (0.04)	0.96 (0.26)	0.93 (0.08)	0.439
CFR	2.8 (1.5)	6.0 (3.3)	2.8 (2.1)	3.9 (1.5)	** < 0.001**
IMR	23 (8)	15 (6)	23 (19)	14 (5)	**0.002**
ACh max dose	0–0–1–3–29	0–0-0–5–10	0–0–2–8–14	1–1-0–3–18	0.060
2 ug	0 (0%)	0 (0%)	0 (0%)	1 (4%)	0.400
20 ug	1 (3%)	0 (0%)	2 (8%)	0 (0%)	0.521
100 ug	3 (9%)	5 (33%)	8 (33%)	3 (13%)	0.056
200 ug	29 (85%)	10 (67%)	14 (58%)	18 (78%)	0.065
**Pharmacotherapy**					
Beta-blocker	19 (56%)	1 (7%)	6 (25%)	10 (43%)	**0.005**
CCB DHP	14 (41%)	1 (7%)	4 (17%)	12 (52%)	**0.005**
CCB nDHP	13 (38%)	13 (87%)	18 (75%)	9 (39%)	**0.001**
Nitrates	4 (12%)	3 (20%)	3 (13%)	3 (13%)	0.891
Statin	31 (91%)	15 (100%)	21 (88%)	20 (87%)	0.543
ACE inhibitors	19 (56%)	9 (60%)	11 (46%)	14 (61%)	0.730
ARB	5 (15%)	0 (0%)	4 (17%)	4 (17%)	0.407
ASA	24 (71%)	10 (67%)	21 (88%)	17 (74%)	0.382
Diuretics	7 (21%)	2 (13%)	6 (25%)	9 (39%)	0.301
MRA	3 (9%)	1 (7%)	2 (8%)	2 (9%)	1.000
Trimetazidine	3 (9%)	2 (13%)	0 (0%)	0 (0%)	0.088

*CMD* coronary microvascular dysfunction, *VSA* vasospastic angina, *BMI* body mass index, *ACS* acute coronary syndrome, *CCS* chronic coronary syndrome, *NYHA* New York Heart Association, *SPECT* single-photon emission computed tomography, *MR* magnetic resonance, *GFR* glomerular filtration rate, *BNP* B-type natriuretic peptide, *LDL-C* low-density lipoprotein cholesterol, *LVEF* left ventricle ejection fraction, *LVEDD* left ventricle end-diastolic diameter, *IVS* interventricular septal thicknesses, *PWT* posterior wall thicknesses, *LVEDP* left ventricle end-diastolic pressure, *LAD* left anterior descending artery, *RFR*, *FFR* fractional flow reserve, *CFR* coronary flow reserve, *IMR* index of microcirculatory resistance, *ACh* acetylcholine, *CCB* calcium channel blocker, *DHP* dihydropyridine, *nDHP* nondihydropyridine, *ACE* angiotensin-converting enzyme, *ARB* angiotensin receptor blockers, *ASA* acetylsalicylic acid, *MRA* mineralocorticoid receptor antagonist

### Concentrations of Different EV Subtypes in Patients with and without INOCA

Patients with INOCA had lower ratio of endothelial EVs exposing VE cadherin (CD144 +) to total EVs, compared to patients with non-anginal chest pain (*p* = 0.027). The ratio of CD144-exposing EVs to total EVs < 0.00016 allowed to diagnose INOCA with 70% sensitivity and 59% specificity (Table 2). In multivariable analysis, only the ratio of CD144 + EVs to total EVs was an independent diagnostic predictor of INOCA (OR 3.38, 95% CI 1.03–11.09, *p* = 0.045) (Table 3).

**Table 2:** Statistical estimates for diagnosis of ischemia and non-obstructive coronary artery disease (INOCA) by the ratio of CD144-exposing extracellular vesicles (EVs) to total EVs, based on the receiver operating characteristic analysis

	AUC (95% CI)	p-value	Cut-off	Sensitivity	Specificity
CD144^+^/total EVs	0.65 (0.53–0.77)	0.027	< 0.00016	70%	59%

*AUC* area under the curve, *CI* confidence interval

**Table 3:** Results of multivariable analysis to diagnose ischemia and non-obstructive coronary artery disease (INOCA) by the ratio of CD144-exposing extracellular vesicles (EVs) to total EVs above the cut-off value and clinical variables

	OR	95% CI		p-value
		Lower	Upper	
CD144^+^/total EVs < 0.00016	3.38	1.03	11.09	**0.045**
BMI, kg/m^2^	0.96	0.85	1.09	0.567
NYHA class	0.64	0.38	1.08	0.096
Beta-blocker	0.76	0.20	2.84	0.685
CCB	0.50	0.14	1.85	0.300

*CI* confidence interval, *OR* odds ratio, *BMI* body mass index, *CCB* calcium channel blocker, *NYHA*—New York Heart Association

**Fig. 2:**
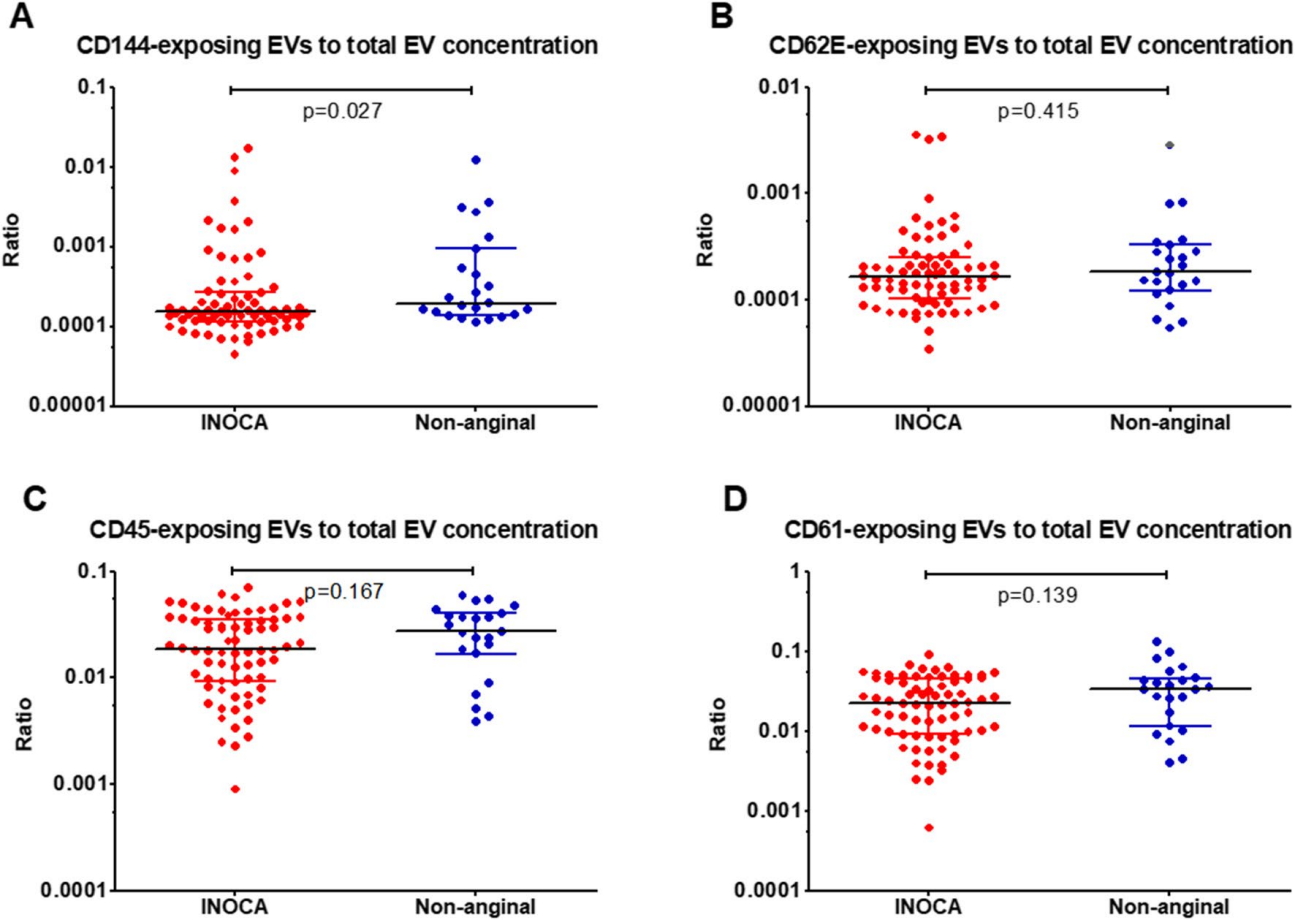
Concentrations of different extracellular vesicles (EV) subtypes (with a diameter exceeding 200 nm in patients with and without ischemia and non-obstructive coronary artery disease (INOCA), expressed as a ratio to total EV concentration and displayed using a logarithmic axis to facilitate perception. **A**: Ratio of CD144-exposing EVs to total EV concentration. **B**: Ratio of CD62E-exposing EVs to total EV concentration. **C**: Ratio of CD45-exposing EVs to total EV concentration. **D**: Ratio of CD61-exposing EVs to total EV concentration. Number of patients: 96. Data are presented as median with interquartile range and compared using U-Mann Whitney test. CD – cluster of differentiation

The ratio of endothelial EVs exposing E-selectin (CD62E +), leucocyte EVs (CD45 +) and platelet EVs (CD61 +) were comparable between the groups (Fig. 2). The total EV concentrations were comparable in patients with INOCA and non-anginal chest pain.

### Concentrations of different EV subtypes depending on the INOCA endotype

**Fig. 3:**
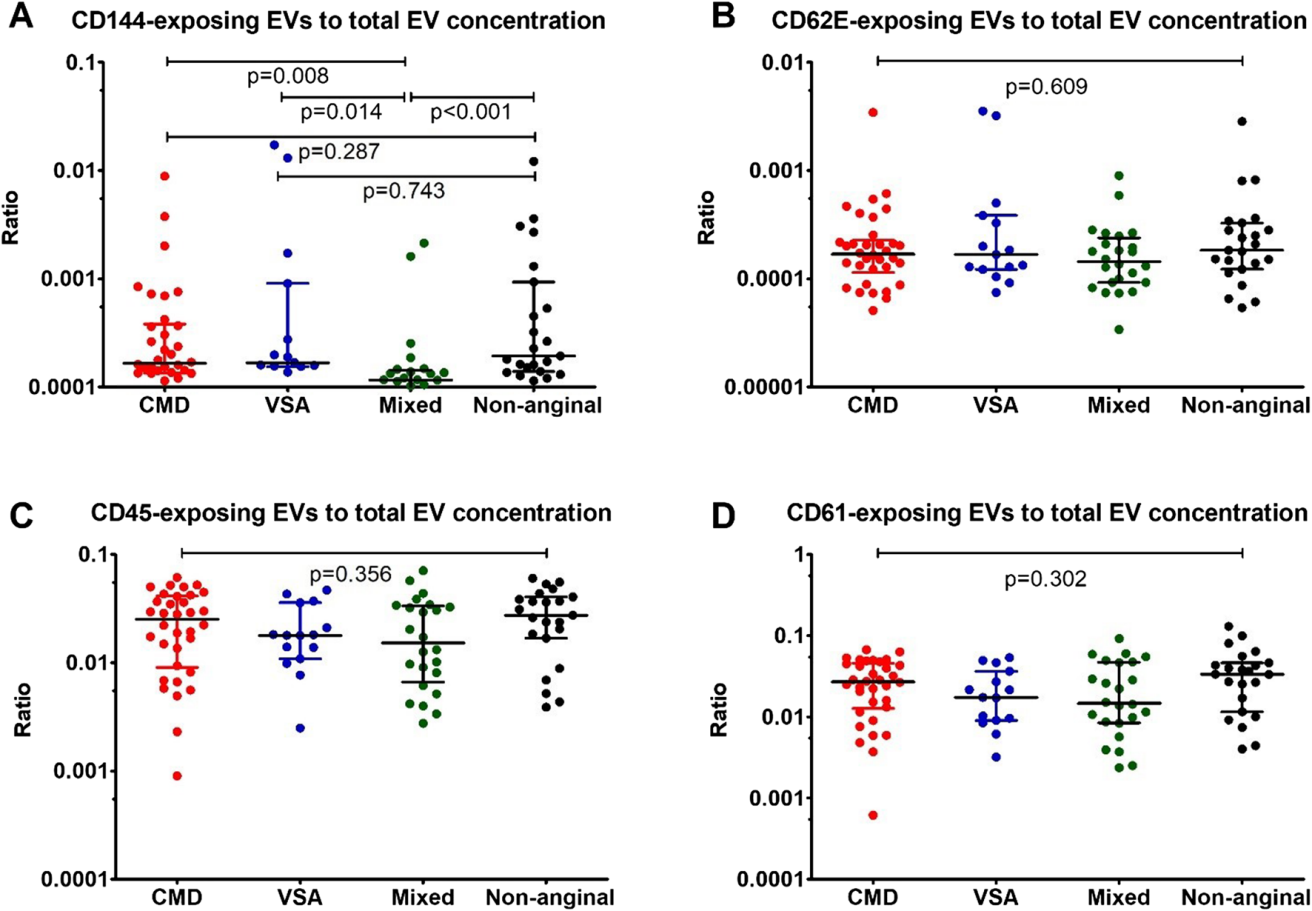
Concentrations of different extracellular vesicles (EV) subtypes with a diameter exceeding 200 nm depending on the endotype of ischemia and non-obstructive coronary artery disease (INOCA), expressed as ratio to total EV concentration and displayed using a logarithmic axis to facilitate perception. **A**: Ratio of CD144-exposing EVs to total EV concentration. **B**: Ratio of CD62E-exposing EVs to total EV concentration. **C**: Ratio of CD45-exposing EVs to total EV concentration. **D**: Ratio of CD61-exposing EVs to total EV concentration. Number of patients: 96. Data are presented as median with interquartile range and compared using Kruskal–Wallis test. CD – cluster of differentiation

Patients with mixed INOCA endotype (CMD + VSA) had lower ratio of endothelial EVs exposing VE cadherin (CD144 +) to total EVs, compared to patients with CMD only (*p* = 0.008), VSA only (p = 0.014) and patients with non-anginal pain (*p* < 0.001). The ratio of endothelial EVs exposing E-selectin (CD62E +), leucocyte EVs (CD45 +) and platelet EVs (CD61 +) were comparable between all four subgroups (Fig. 3).

### Total plasma EV Concentrations

**Fig. 4:**
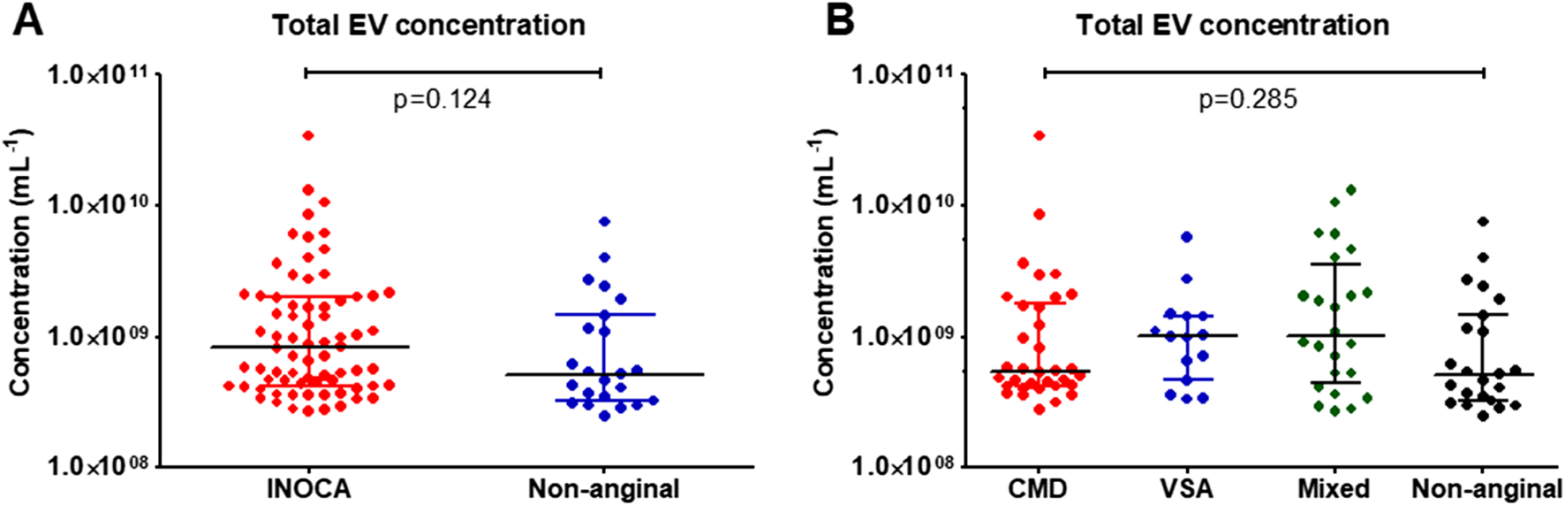
**A**: Total plasma concentrations of extracellular vesicles (EV) with a diameter exceeding 200 nm in patients with and without ischemia and non-obstructive coronary artery disease (INOCA). **B**. Total plasma concentrations of extracellular vesicles (EV) with a diameter exceeding 200 nm depending on the endotype of INOCA. Number of patients: 96. Data are presented as median with interquartile range and compared using Kruskal–Wallis test

To ensure that the observed differences are not due to the differences in total EV concentrations in the measured plasma samples from different patient subgroups, we compared the total EV concentrations in patients with INOCA and non-anginal chest pain and with the different INOCA endotypes. There were no significant differences between the total plasma EV concentrations between the groups (Fig. 4).

### Correlations between EVs, Echocardiographic Parameters and BNP

There were weak, positive correlations between BNP and the ratio of endothelial EVs exposing E-selectin (CD62E +) to total EVs (r = 0.204, *p* = 0.037), as well as the ratio of leukocyte EVs (CD45 +) to total EVs (r = 0. 256, *p* = 0.025). There were weak, negative correlations between left ventricle ejection fraction (LVEF) and the ratio of leukocyte EVs (CD45 +) to total EVs (r = 0.210, *p* = 0.047), as well as the ratio of platelet EVs (CD61 +) to total EVs (r = 0. 228, *p* = 0.031). There were no other significant correlations between plasma EV concentrations and echocardiographic parameters or BNP (Table 4).

**Table 4:** Correlations between extracellular vesicles (EVs), echocardiographic parameters and B-type natriuretic peptide (BNP). Significant correlations are made bold and marked with a star. * *p* < 0.05, **p < 0.01, ***p < 0.001

	LVEF, %	LVEDD, mm	IVS, mm	PWT, mm	BNP, pg/ml
CD144^+^/total EVs	−0.010	−0.204	0.030	0.122	0.40
CD62E^+^/total EVs	−0.056	−0.196	−0.045	0.001	0.240*
CD45^+^/total EVs	−0.210*	0.090	0.013	0.083	0.256*
CD61^+^/total EVs	−0.228*	0.039	−0.059	0.081	0.206

*BNP* B-type natriuretic peptide, *LVEF* left ventricle ejection fraction, *LVEDD* left ventricle end-diastolic diameter, *IVS* interventricular septal thicknesses, PWT

## Discussion

To our best knowledge, this is the first prospective, multicenter study investigating the differences in EV concentrations in patients with various INOCA endotypes. The main findings are that (i) patients with INOCA have lower ratio of endothelial EVs (CD144 +) to total EV concentration, and (ii) patients with combined CMD and VSA have lower ratio of endothelial EVs (CD144 +) to total EV concentration, compared to all other investigated subgroups: patients with only CMD, only VSA and non-anginal chest pain.

VE-cadherin (CD144) is an adhesion molecule located at junctions between endothelial cells, constitutively expressed and specific for endothelial cells [20]. Increased concentrations of EVs exposing VE-cadherin were found in patients with obstructive CAD, chronic heart failure, acute ischemic stroke and heart transplant recipients with graft vasculopathy, compared to healthy controls [13]. In contrast, we found that patients with mixed INOCA endotype (CMD + VSA) had lower ratio of EVs exposing VE-cadherin to total EV, compared to other INOCA endotypes and patients with non-anginal pain. From the pathophysiological perspective, the co-existence of CMD and VSA requires the combination of enhanced coronary vasoconstriction and impaired coronary vasodilation [21]. Endothelial cell apoptosis is the key event in both CMD and VSA, and VE-cadherin was found to have an anti-apoptotic effect by enhancing endothelial cell proliferation [22]. Hence, our results imply that a lower ratio of EVs exposing VE-cadherin to total EVs might reflect impaired VE-cadherin-mediated anti-apoptotic effect. This phenomenon would lead to more endothelial cell apoptosis, manifesting as the most severe INOCA endotype. To support this hypothesis, patients with mixed INOCA endotype seem to have more aggravated symptoms and worse overall quality of life compared with other endotypes [1, 7].

The management of the mixed INOCA endotype is challenging, since it requires the combination of calcium channel blockers, nicorandil and/or third line agents such as ranolazine or trimetazidine [7]. However, these therapies mainly focus on the vasospastic component and are based on expert opinion, as no specific trials have hitherto focused on patients with mixed INOCA endotype. Based on our findings suggesting impaired anti-apoptotic mechanisms, reflected by lower ratio of EVs exposing VE-cadherin to total EVs, it could be hypothesized that tailored therapies to specifically inhibit microvascular endothelial cell apoptosis might improve symptoms in this subgroup of patients. Such antiapoptotic properties have been attributed to nitric oxide donors (L-arginine, antioxidative vitamins), N-acetylcysteine, metformin or sulodexide [23]. Although better known for its antithrombotic effect, sulodexide endothelial-protective properties were proven beneficial in numerous diseases with impaired endothelial function, including chronic venous disease, peripheral arterial disease, Raynaud syndrome, diabetic retinopathy and early stages of COVID-19 [24–26]. Whether sulodexide might improve symptoms and quality of life in patients with mixed INOCA endotype remains an open research question.

We did not find any differences in plasma concentrations of endothelial EVs exposing E-selectin, platelet and leukocyte EVs between INOCA patients and patients with non-anginal pain, and between different INOCA endotypes. This might be because EVs exposing E-selectin are not constitutively expressed on endothelial cells, but rapidly up-regulated in response to inflammation [13]. Similarly, platelet and leukocyte EVs are increased mostly in atherosclerotic-related and inflammatory diseases [14, 27]. The correlations between heart failure parameters (BNP and LVEF) and endothelial EVs exposing E-selectin, leukocyte EVs, and platelet EVs might imply that patients with more advanced heart failure, which is known to be associated with systemic pro-inflammatory response, have relatively higher relative concentrations of these EVs. Nevertheless, in our hands, the release of these EV subtypes did not reflect the presence of INOCA or the specific endotype, likely due to their lack of specificity for endothelial dysfunction.

## Strengths and Limitations

The novelty of the project is the first ever attempt to identify a “circulating footprint" indicating the specific INOCA endotype based on plasma EV concentrations. Compared to previous studies, we used (i) a standardized definition of CMD and VSA based on an interventional coronary function testing and (ii) reliable and reproducible assessment of EVs concentration using dedicated infrastructure, software and standardized reporting of the results (MIFlowCyt-EV). There are limitations of this study, which should be acknowledged too.

First, the primary endpoint of the study enabled to differentiate INOCA from non-anginal chest pain, whereas the number of patients per subgroups was too low to derive firm conclusion regarding the diagnostic utility of the identified biomarker to differentiate between INOCA endotypes. The low number of patients per subgroup was due to two reasons (i) the preliminary character of our study, and (ii) the sample size calculation for the difference in EV concentrations between patients with any INOCA endotype and non-anginal chest pain (primary endpoint), but not between the subgroups of INOCA. Considering the promising results of our study, future studies should be powered to detect differences between various INOCA endotypes, before EVs can be used as clinically applicable biomarkers. Second, we did not show any mechanistic relationship between the decreased ratio of endothelial EVs (CD144 +) to total EVs and the pathophysiology of mixed INOCA endotype (CMD + VSA). Further basic research studies are required to elucidate the role of various EV subtypes in the pathogenesis of INOCA. Third, we collected blood only once, so we cannot exclude that there are certain variations in EV concentration over time. Hence, before EVs might be used as biomarkers, temporal changes in EV concentrations and the dynamics of their release and clearance should be established. Fourth, we did not evaluate the effect of the concomitant treatment on EV concentrations, which might have affected the results as well. However, in multivariable analysis, only the ratio of CD144 + EVs to total EVs was an independent diagnostic predictor of INOCA, whereas the treatment with beta blockers and calcium channel blockers did not affect the diagnosis. Altogether, the results and conclusions should be considered as hypothesis-generating, interpreted with caution and confirmed in future studies.

## Outlook and Conclusions

Despite accumulating data regarding the molecular properties and impact of EV subtypes in vascular homeostasis, the utility of EVs as non-invasive biomarkers in cardiovascular diseases is still being extensively studied. We found that patients with combined CMD and VSA have significantly lower ratio of endothelial EVs (CD144 +) to total EVs, compared to patients with only CMD, only VSA and non-anginal chest pain. We propose a "signature" of mixed INOCA endotype based on the measurement of CD144-exposing EV concentration. The next step is to conduct a larger trial focusing on endothelial EVs as novel biomarkers in INOCA. Such biomarkers would have the potential to revolutionize INOCA diagnostic algorithm, allowing to eliminate the need for invasive coronary function testing.

## Supplementary Information

Below is the link to the electronic supplementary material.

Supplementary file1 (DOCX 1737 KB)

## Data Availability

The datasets generated and analysed during the current study are available from the corresponding author on reasonable request.
