# Supplementary material for: Endothelial Cell-Derived Extracellular Vesicles Allow to Differentiate Between Various Endotypes of INOCA: A Multicentre, Prospective, Cohort Study
Source: J Cardiovasc Transl Res. 2024 Dec 5;18(2):305–15. doi: 10.1007/s12265-024-10575-x (PMC12043753; doi:10.1007/s12265-024-10575-x)
Supplement: Supplementary file 1 — Supplementary file1 (DOCX 1737 KB) [file 12265_2024_10575_MOESM1_ESM.docx]

MIFlowCyt-EV of study “INOCA”

This document aims to provide the minimum information required to reproduce the flow cytometry experiments on extracellular vesicles (EVs) performed in the study “INOCA”. This document is based on three published standardization frameworks and guidelines [1–3].

**Contents**

[1 Experiment overview 2](#_Toc155089779)

[1.1 Contact details 2](#_Toc155089780)

[1.1.1 Experiment leader 2](#_Toc155089781)

[1.1.2 Flow cytometry execution 2](#_Toc155089782)

[1.2 Purpose 2](#_Toc155089783)

[1.3 Keywords 2](#_Toc155089784)

[1.4 Experiment variables 2](#_Toc155089785)

[1.5 Experiment design and quality controls 2](#_Toc155089786)

[1.6 Dates 3](#_Toc155089787)

[1.7 Conclusions 3](#_Toc155089788)

[2 Sample details 4](#_Toc155089789)

[2.1 Sample description 4](#_Toc155089790)

[2.1.1 Sample source description 4](#_Toc155089791)

[2.1.2 Sample description 4](#_Toc155089792)

[2.2 Sample collection 4](#_Toc155089793)

[2.3 Sample storage 4](#_Toc155089794)

[2.4 Sample characteristics 4](#_Toc155089795)

[2.5 Sample dilution 4](#_Toc155089796)

[2.6 Sample staining 5](#_Toc155089797)

[2.7 Fluorescence reagents 6](#_Toc155089798)

[3 Flow cytometer 7](#_Toc155089799)

[3.1 Model and manufacturer 7](#_Toc155089800)

[3.2 Configuration and settings 7](#_Toc155089801)

[3.2.1 Flow rate and acquisition time 7](#_Toc155089802)

[3.2.2 Light sources 7](#_Toc155089803)

[3.2.3 Detectors 7](#_Toc155089804)

[3.2.4 Trigger detector and threshold 7](#_Toc155089805)

[4 Assay controls 8](#_Toc155089806)

[4.1 Buffer-only controls 8](#_Toc155089807)

[4.2 Buffer with reagents controls 8](#_Toc155089808)

[4.3 Unstained controls 8](#_Toc155089809)

[4.4 Isotype controls 8](#_Toc155089810)

[4.5 Detergent treatment controls 9](#_Toc155089811)

[5 Data analyses 9](#_Toc155089812)

[5.1 Data sharing 9](#_Toc155089813)

[5.2 Compensation details 9](#_Toc155089814)

[5.3 Calibrations 9](#_Toc155089815)

[5.3.1 Flow rate 9](#_Toc155089816)

[5.3.2 Fluorescence calibration 10](#_Toc155089817)

[5.3.3 Light scattering calibration 11](#_Toc155089818)

[5.4 Gate description and boundaries 12](#_Toc155089819)

[6 References 13](#_Toc155089820)

# Experiment overview

## Contact details

### Experiment leader

| Name of organization | Medical University of Warsaw |
| --- | --- |
| Address | Ulica Żwirki i Wigury 61  02-091 Warsaw  Poland |
| Primary contact name | Dr. Aleksandra Gąsecka – van der Pol |
| Primary contact e-mail address | [aleksandra.gasecka@wum.edu.pl](mailto:aleksandra.gasecka@wum.edu.pl) |

### Flow cytometry execution

| Name of organization | EV Count, Amsterdam University Medical Centers |
| --- | --- |
| Address | Meibergdreef 9  PO Box 22660  1100 DD  Amsterdam  The Netherlands |
| Contact name | Britta Bettin |
| Contact e-mail address | [b.bettin@evcount.com](mailto:b.bettin@evcount.com) |

## Purpose

The goal of this flow cytometry experiment is to investigate whether coronary microvascular dysfunction can be differentiated from vasospastic angina based on the concentration of EVs from endothelial cells and vascular smooth muscles cells in human blood plasma. The hypothesis is that concentrations of EVs from endothelial cells and vascular smooth muscles cells in plasma differ between patients with coronary microvascular dysfunction and vasospastic angina.

## Keywords

Extracellular vesicles, flow cytometry, human blood plasma, coronary microvascular dysfunction, vasospastic angina, endothelial cells, vascular smooth muscles cells

## Experiment variables

The experimental variable is whether patients were diagnosed with either coronary microvascular dysfunction or vasospastic angina. In total, 96 patients were included in the analysis: 34 with CMD (35%), 15 with VSA (16%), 24 with mixed endotype (25%) and 23 with non-anginal chest pain (24%) (healthy controls).

## Experiment design and quality controls

All samples were measured using an autosampler, which facilitates subsequent measurements of samples in a 96-well plate. The entire study involved four 96-well plates that were measured within 1 month. Each well plate contained a buffer-only control, reagents in buffer controls, detergent lysis control and isotype controls. Flow rate, fluorescence, and light scattering calibrations were performed daily.

## Dates

Samples were collected between 11 September 2021 and 4 April 2023 in the 1^st^ Chair and Department of Cardiology, Medical University of Warsaw, Poland and Department of Interventional Cardiology, Institute of Cardiology, Jagiellonian University Medical College, Krakow, Poland. Flow cytometry experiments were performed between 25 July 2023 and 28 August 2023 by EV Count.

## Conclusions

We found that patients with combined CMD and VSA have significantly lower ratio of endothelial EVs (CD144+) to total EVs, compared to patients with only CMD, only VSA and non-anginal chest pain. We propose a "signature" of mixed INOCA endotype based on the measurement of CD144-exposing EV concentration. The next step is to conduct a larger trial focusing on endothelial EVs as novel biomarkers in INOCA. Such biomarkers would have the potential to revolutionize INOCA diagnostic algorithm, allowing to eliminate the need for invasive coronary function testing.

# Sample details

## Sample description

### Sample source description

Hospitalized patients with CMD or VSA. Graphical Abstract contains the inclusion criteria. Table 1 contains an overview of the patient characteristics.

### Sample description

Intravenously collected blood.

## Sample collection

Blood collected in 7.5 mL 0.109 mol/L ethylenediaminetetraacetic acid (EDTA) plastic tubes (S-Monovette, Sarstedt, Germany) via antecubital vein puncture using a 21-gauge needle, without tourniquet. The first 2 mL of collected blood were discarded to avoid that activation of endothelial cells and platelets during vein puncture would affect the measurement results.

## Sample storage

Within 1 hour from blood collection, plasma was prepared by double centrifugation using a Rotina 380 R equipped with a swing-out rotor and a radius of 155 mm (Hettich Zentrifugen, Tuttlingen, Germany). The centrifugation parameters were: 2,500 g, 15 minutes, 20˚C, acceleration speed 1, no brake. The first centrifugation step was done with 7.5 mL whole blood collection tubes. Supernatant was collected 10 mm above the buffy coat. The second centrifugation step was done with 3.5 mL plasma in 15 mL polypropylene centrifuge tubes (Greiner Bio-One, Austria). Supernatant was collected 5 mm above the buffy coat, transferred into 5 mL polypropylene centrifuge tubes (Greiner Bio-One, Austria), mixed by pipetting, aliquoted in volumes of 250 µL, transferred to 1.5 mL low-protein binding Eppendorfs (Thermo Fisher Scientific, US) and stored in -80˚C.

## Sample characteristics

Frozen human plasma samples are expected to contain the following particles: erythrocyte ghosts, EVs, lipoproteins, platelets, precipitated salt crystals, proteins, and complexes of the aforementioned particles.

## Sample dilution

As the concentration of particles in plasma differs >10^2^-fold between donors, samples require different dilutions to (1) avoid swarm detection and (2) detect a statistically significant number of events within a measurement time of a few minutes. The optimal dilution factor is the minimum dilution factor that is required to prevent swarm detection. For the flow cytometer and settings used, the optimal dilution factor for plasma is ≥1.1∙10^2^ -fold and should result in a count rate <1.1∙10^4^ events∙s^-1^ [4].

To find the dilution resulting in a count rate <1.1∙10^4^ events∙s^-1^, we diluted each sample 1,000‑fold in Dulbecco′s phosphate buffered saline (DPBS) and measured the total concentration of particles for 30 s without staining. For all experiments, filtered DPBS (Corning, US) was used. By diluting each sample 1,000-fold, all samples had a count rate <1.1∙10^4^ events∙s^-1^. Figure 1A shows a distribution of the measured total particle concentrations of all samples in the study. Taking into account the measured concentration and flow rate, we calculated the minimum dilution factor required before staining (section 2.6) to achieve a count rate <1.1∙10^4^ events∙s^-1^ after staining. The staining procedure adds an extra dilution factor of 11.1-fold to the overall dilution. To simplify the pipetting procedures, samples were divided into 6 categories of pre-staining dilution factors: 10-fold, 16-fold, 25-fold, 40-fold, 65-fold and 160-fold. Figure 1B shows a distribution of the applied pre-staining dilution factors of all samples in the study.


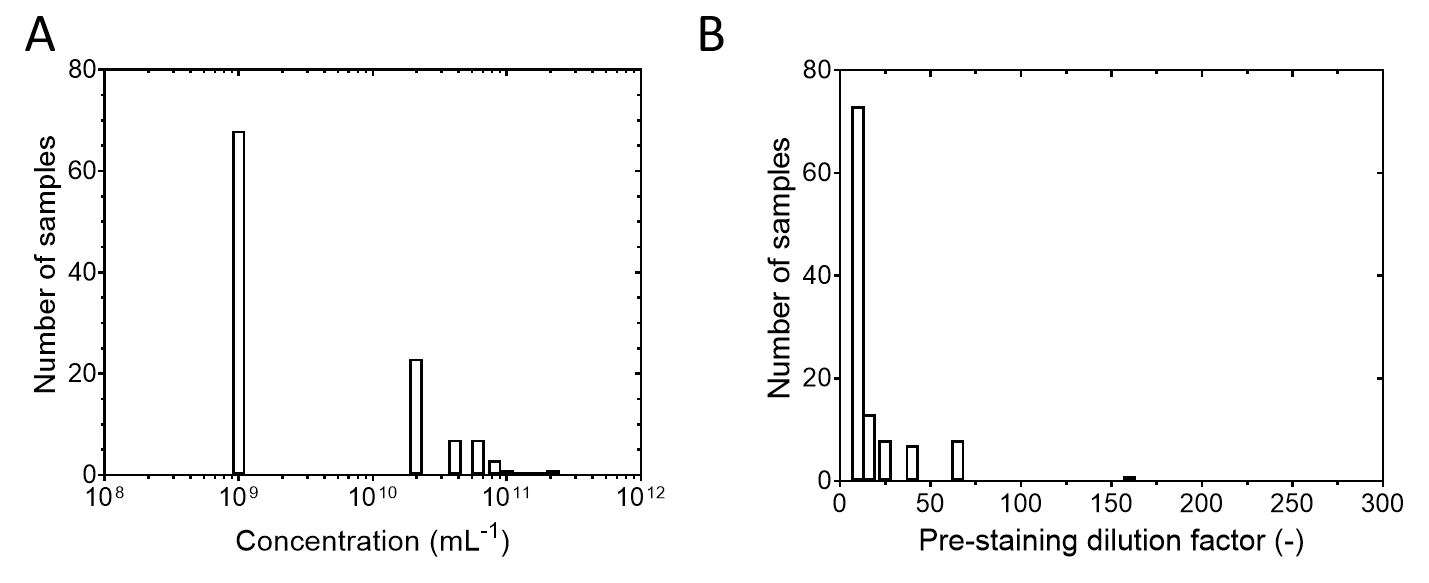


Figure 1: (A) Distribution of the total concentration of particles exceeding the trigger threshold for all samples in the study. (B) Distribution of the applied pre-staining dilution factors for all samples in the study.

## Sample staining

Table 1 shows an overview of the antibodies that were used to stain EVs in plasma. Prior to staining, the antibodies were diluted in DPBS. For each antibody the optimal dilution factor was determined by titration (Table 1). To remove aggregates, diluted antibodies were centrifuged at 18,890 g for 5 min at 20 °C. The supernatant minus 10 μL of the starting volume was collected and used for staining. Each sample was double stained with CD45-APC (allophycocyanin) and CD62E-PE (phycoerythrin), with CD61-APC and CD144-PE. To stain, 20 μL of pre-staining diluted (Figure 1B) plasma was incubated with 2.5 μL of each antibody or isotype controls and kept in the dark for 2 h at room temperature. After the incubation, samples were diluted in 200 μL DPBS to decrease background fluorescence from unbound reagents.

## Fluorescence reagents

| **Characteristic**  **measured** | **Analyte** | **Analyte detector** | **Reporter** | **Isotype** | **Clone** | **Concentration (µg mL^-1^)** | **Manufacturer** | **Catalog number** | **Lot number** | **Dilution factor** |
| --- | --- | --- | --- | --- | --- | --- | --- | --- | --- | --- |
| Integrin | Human  CD61 | Anti-human CD61 antibody | APC | IgG1 | VI-PL2 | 50 | eBioscience™ | 17-0619-42 | 2062626 | 64x |
| Adhesion molecule | Human  CD62E | Anti-human CD62E antibody | PE | **IgG1** | 68-5H11 | 100 | BD Pharmingen™ | 551145 | 2265697 | 16x |
| Adhesion molecule | CD144 | Anti-human CD144 antibody | PE | IgG1 | 55-7H1 | 3 | BD Pharmingen™ | 560410 | 2182431 | 2x |
| Leukocyte common antigen | CD45 | Anti-human CD45  antibody | APC | IgG1 | HI30 | 9 | Biolegend | 304037 | B311564 | 8x |
| Affinity for Fc receptor | Fc receptor | IgG1 κ | APC | n/a | MOPC-21 | 200 | BD Pharmingen™ | 554681 | 0030904 | 178x |
|  | Fc receptor | IgG1 | PE | n/a | X40 | 50 | BD Pharmingen™ | 9309643 | 7248665 | 8x |

Table 1. Overview of staining reagents. Characteristics being measured, analyte, analyte detector, reporter, isotype, clone, concentration during staining, manufacturer, catalog number and lot number of used staining reagents. The concentration of staining reagents during measurements was 11.1-fold lower than the concentration during staining. APC: allophycocyanin; CD: cluster of differentiation; FITC: fluorescein isothiocyanate; IgG: immunoglobulin G; PE: phycoerythrin; BD: Becton Dickinson.

# Flow cytometer

## Model and manufacturer

A60-Micro, Apogee Flow Systems, Hemel Hempstead, UK. The flow cytometer has not been altered. All components are original and came with the flow cytometer.

## Configuration and settings

### Flow rate and acquisition time

The flow cytometer is equipped with a syringe pump with volumetric control. Samples were analysed for 240 s at a flow rate of 3.01 μL/min.

### Light sources

The flow cytometer has three lasers that illuminate a fixed-alignment cuvette flow cell. The laser powers were 100 mW, 150 mW and 150 mW for the 405-nm, 488-nm, and 638-nm laser, respectively.

### Detectors

Table 2 shows an overview of the detectors used in this study.

| Detector name | Detected property | Voltage (V) | Spectral filter bandwidth (nm) |
| --- | --- | --- | --- |
| 405-SALS | Forward scattered light | 470 |  |
| 405-LALS | Side scattered light | 375 |  |
| 488-Orange | PE fluorescence | 450 | 575/30 |
| 638-D Red | APC fluorescence | 480 | >650 |

Table 2. Detector name, detected property, voltage and spectral filter bandwidth of the detectors used in this study. APC: allophycocyanin; PE: phycoerythrin; SALS: small angle light scattering; LALS: large angle light scattering.

### Trigger detector and threshold

Based on the buffer-only control, a trigger threshold of 24 arbitrary units was applied to the side scattering detector. As (1) the A60-Micro applies the trigger threshold analogically, thus before digitalization of the signal, and (2) the arbitrary unit channel numbers differ between the data acquisition software and the flow cytometry datafiles, the trigger threshold was expressed in standard units by taking the mode of the effective side scattering cross section distribution and optical diameter distribution (section 5.3.3.1) of a plasma sample. Plasma samples have a size distribution with a peak far below the detection limit of the A60-Micro. The modes of the effective side scattering cross section distribution and optical diameter distribution therefore represent the trigger threshold. The trigger threshold is equivalent to an effective side scattering cross section of 10 nm^2^ and an optical diameter of 165 nm for EVs^[[1]](#footnote-1)^.

# Assay controls

Assay controls recommended by the MIFlowCyt-EV framework were performed to confirm that signals originate from EVs. Fluorescence-minus-one and single-stained controls were not performed due to thorough experience with the used antibody panels and because the emission spectra of the used fluorophores do not have spectral overlap. Procedural controls were not performed because no methods to isolate EVs were applied after staining. Serial dilution control were performed on the A60-Micro for six representative plasma samples and results were published [4]. Section 2.5 explains how swarm detection was prevented.

## Buffer-only controls

Each 96-well plate contained at least 1 well with DPBS, which was measured with the same flow cytometer and acquisition settings as all other samples. The median count rate for all DPBS measurements was 17 events s^-1^, which is lower than the target count rate (7.0∙10^3^ events s^-1^) for events in stained plasma samples.

## Buffer with reagents controls

Each 96-wellplate contained a buffer with reagent control for each reagent (Table 1), which was

measured with the same flow cytometer and acquisition settings as all other samples. Table 3 shows a summary of the results of the buffer with reagents controls.

| Reagent | Mean number of fluorescence positive events in buffer  (240 s^-1^) | Mean number of fluorescence positive events in stained samples  (240 s^-1^) | Mean number of fluorescence positive events in buffer / stained samples (-) |
| --- | --- | --- | --- |
| CD61-APC | 7 | 3714 | 0.0019 |
| CD45-APC | 14 | 2006 | 0.0070 |
| CD62E-PE | 16 | 69 | 0.2310 |
| CD144-PE | 25 | 129 | 0.1924 |

Table 3. Results of the buffer with reagents controls. APC: allophycocyanin; CD: cluster of differentiation; PE: phycoerythrin.

## Unstained controls

Unstained controls were measured with a 1,000-fold dilution factor, which differs from the dilution factor with which the stained plasma samples were measured. Unstained controls were not considered in this analysis.

## Isotype controls

Each 96-wellplate contained an arbitrary plasma sample stained with IgG1, which was

measured with the same flow cytometer and acquisition settings as all other samples. Table 4 shows a summary of the results of the isotype controls.

| Sample | Mean number of fluorescence positive events in isotype control (120 s^-1^) | Mean number of fluorescence positive events in stained samples  (120 s^-1^) | Mean number of fluorescence positive events in isotype control / stained samples (-) |
| --- | --- | --- | --- |
| CD45 APC | 10 | 2109 | 0.005 |
| CD62E-PE | 277 | 943 | 1.061 |
| CD61-APC | 10 | 4797 | 0.002 |
| CD144-PE | 277 | 999 | 0.277 |

Table 4. Results of the isotype controls. APC: allophycocyanin; CD: cluster of differentiation; PE: phycoerythrin.

## Detergent treatment controls

Each 96-wellplate contained an arbitrary plasma sample to which 10% NP-40 (Merck Life Science, The Netherlands) was added. After vortexing for 5 seconds, the sample was measured with the same flow cytometer and acquisition settings as all other samples. Table 5 shows a summary of the results of the detergent treatment controls.

| Reagent | Mean number of fluorescence positive events after detergent treatment (120 s^-1^) | Mean number of fluorescence positive events in stained samples  (120 s^-1^) | Mean number of fluorescence positive events after detergent treatment / stained samples (-) |
| --- | --- | --- | --- |
| CD45-APC | 28 | 2067 | 0.013 |
| CD62E-PE | 20 | 47 | 0.426 |

Table 5. Results of the detergent treatment controls. APC: allophycocyanin; CD: cluster of differentiation;PE: phycoerythrin.

# Data analyses

To automatically determine optimal samples dilutions, apply calibrations, determine and apply gates, generate reports with scatter plots and generate data summaries, we developed and applied custom-build software (MATLAB R2020b, Mathworks, USA).

## Data sharing

Data are available upon request to the Corresponding Author, Aleksandra Gąsecka ([gaseckaa@gmail.com](mailto:gaseckaa@gmail.com))

## Compensation details

No compensation was applied because no fluorophore combinations were used that have overlapping emission spectra.

## Calibrations

### Flow rate

At the start of each measurement day, we applied the automated quality control system ApoCal (#1524, Apogee Flow Systems), which checks whether the flow rate is within 20% of the adjusted flow rate of 3.01 µL∙min^-1^. For all days, the flow cytometer passed this quality control check.

### Fluorescence calibration

Calibration of the fluorescence detectors from arbitrary units (a.u.) to molecules of equivalent soluble fluorochrome (MESF) was accomplished using 2 µm APC quantification beads (lot 2364-19, custom-order, Becton Dickinson Biosciences) and 2 µm PE Quantification beads (lot 2364-89, custom-order, Becton Dickinson Biosciences). Figure 2A and B show the 10-base logarithm of the MESF intensities for the MESF beads versus the 10-base logarithm of the measured median fluorescence intensity of each bead population. The data are fitted with a linear function. These fluorescence calibrations were used to assign MESF values for APC and PE to rainbow beads (SPHERO^TM^ Rainbow calibration particles, 8 peaks, 3.0-3.4 µm, lot EAP01, Spherotech). In turn, the rainbow beads, which are hard dyed beads with long-term stability, were used to apply fluorescence calibrations on a daily basis. Figure 2C and D show the 10-base logarithm of the assigned MESF intensities for rainbow beads versus the 10-base logarithm of the measured median fluorescence intensity of each bead population. The data are fitted with a linear function. For each measured plasma sample, we added fluorescent intensities in MESF units to the flow cytometry data files using following equation:

| $\text{I(MESF)}={10}^{a\cdot\log_{10} \text{I(a.u.)}+b}$ | Equation 1 |
| --- | --- |

where I is the fluorescence intensity, and *a* and *b* are the slope and the intercept of the linear fits in Figure 2.


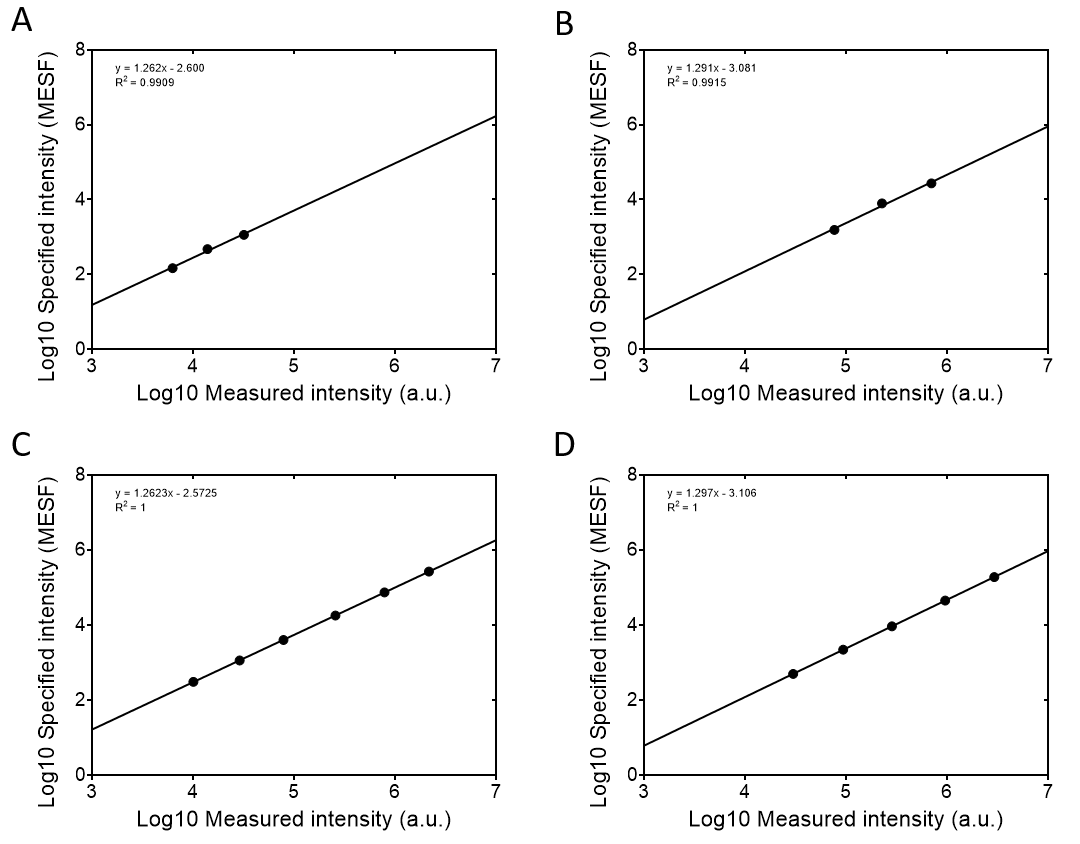


Figure 2. Calibration of the fluorescence detectors from arbitrary units (a.u.) to molecules of equivalent soluble fluorochrome (MESF). Logarithmic MESF versus logarithmic median fluorescence intensity for (A) phycoerythrin (PE), and (B) allophycocyanin (APC). Furthermore, the 10-base logarithm of the assigned MESF intensities for rainbow beads versus 10-base logarithm of the measured median fluorescence intensity of each bead population are shown for PE (C), and APC (D). Data (symbols) are fitted with a linear function (line).

### Light scattering calibration

#### Rosetta Calibration

Rosetta Calibration (v2.05, Exometry, The Netherlands) was used to relate the forward and side scattering intensities measured at a wavelength of 405 nm to the effective scattering cross sections^[[2]](#footnote-2)^ and optical diameter^[[3]](#footnote-3)^ of EVs. EVs are modelled as core-shell particles with a core refractive index of 1.38, a shell refractive index of 1.48, and a shell thickness of 6 nm. Figure 3 shows print screens of the light scatter calibrations.

A B


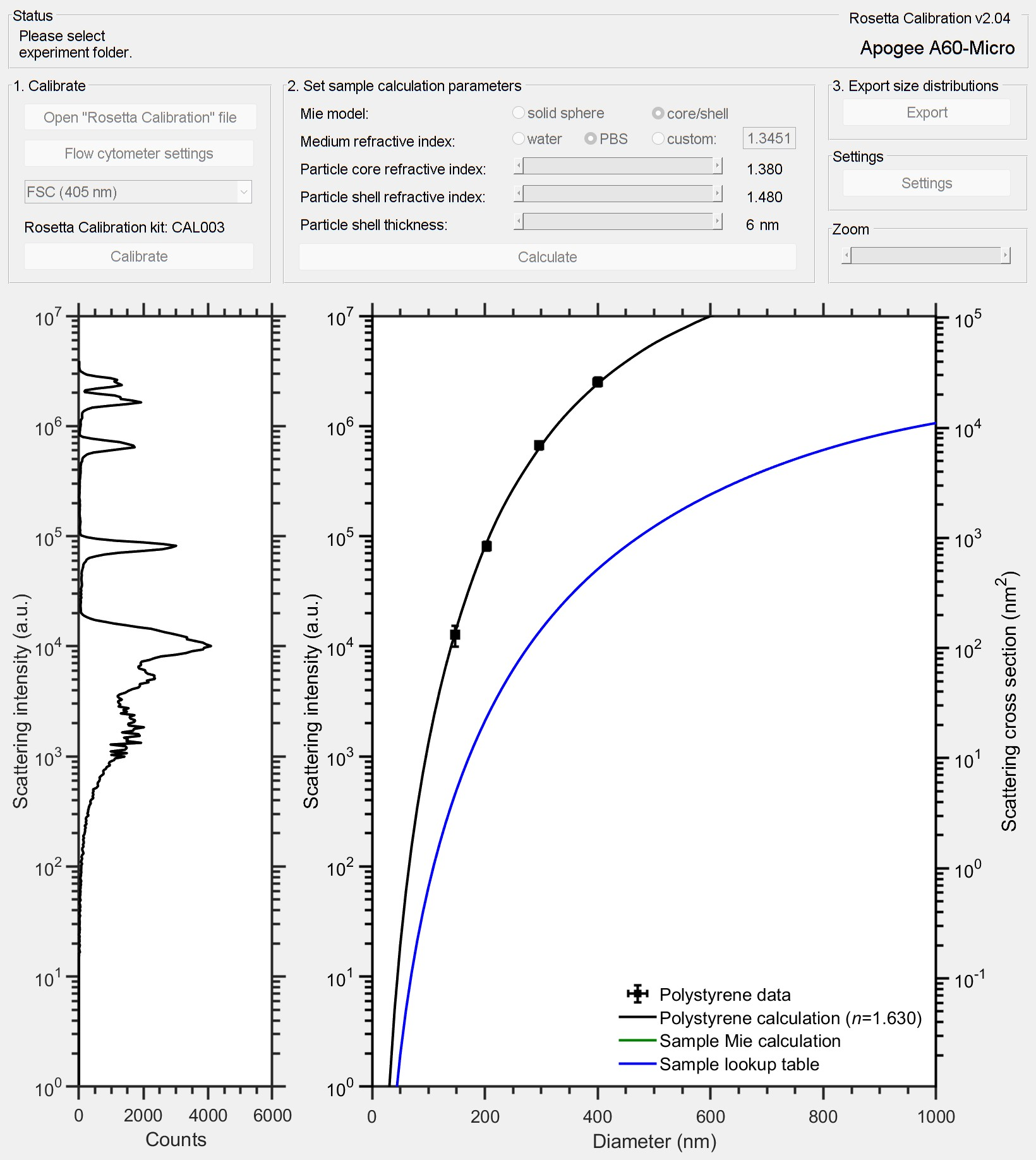

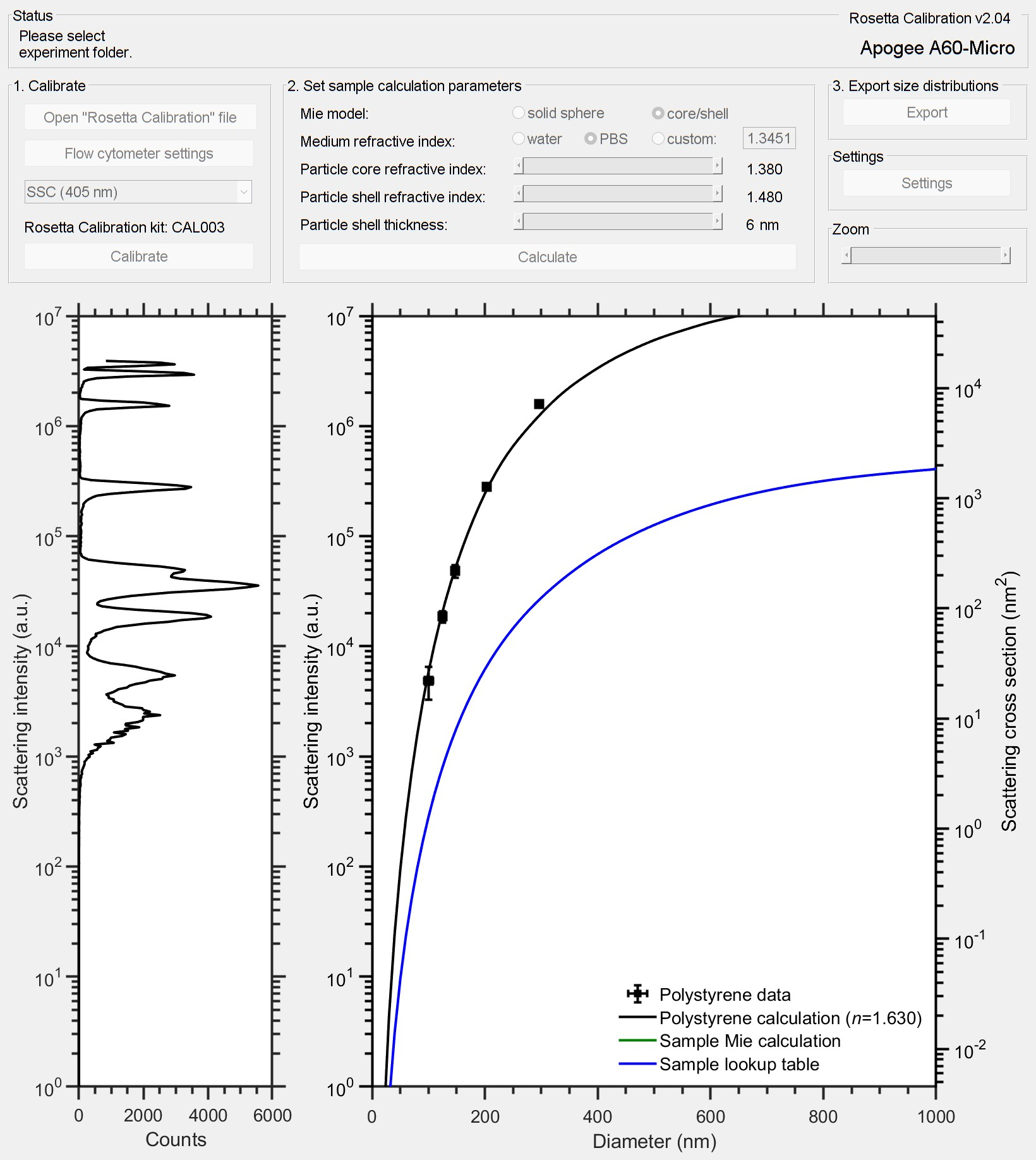


Figure 3. Forward scattering and side scattering calibration of the A60-Micro by Rosetta Calibration. To relate scatter to the diameter of EVs, EVs are modelled as core-shell particles with a core refractive index of 1.38, a shell refractive index of 1.48, and a shell thickness of 6 nm.

#### Flow Scatter Ratio (Flow-SR)

To determine the diameter and refractive index of particles and improve specificity by enabling label-free differentiation between EVs and lipoproteins, the flow scatter ratio (Flow-SR) was applied.

Flow-SR was performed as previously described [5,6]. Lookup tables were calculated for diameters ranging from 10 to 1000 nm, with step sizes of 1 nm, and refractive indices from 1.35 to 1.80 with step sizes of 0.001. The diameter and refractive index of each particle was added to the flow cytometry datafiles by custom-build software (MATLAB R2020b).

As Flow-SR requires accurate measurements of both forward scattering and side scattering, we applied Flow-SR only to particles with diameters >200 nm, as determined by Flow-SR, and fulfilling the condition:

| $\log_{10} \sigma_{SSC}>-0.8\cdot\log_{10} \sigma_{FSC}+2.8$ | Equation 2 |
| --- | --- |

where $\sigma_{SSC}$ is the side scattering cross section and $\sigma_{FSC}$ is the forward scattering cross section.

## Gate description and boundaries

The following gates have been applied to all flow cytometry data files by custom-build software (MATLAB R2020b):

1. During the second that an event was measured, the count rate was within 3.5 standard deviations from the median count rate of the entire measurement, and…
2. the CD45-APC and CD61-APC fluorescence was <1600 MESF or the side scattering cross section was <2800 nm^2^ to omit cells (only applied to samples stained with CD45-APC and CD61-APC), and…
3. the condition in Equation 3 was fulfilled to remove antibody aggregates (only applied to CD62E-PE and CD144-PE antibodies), and…
4. the optical diameter is <1,000 nm, and…
   1. the CD45-APC fluorescence >21 MESF, or…
   2. the CD62E-PE fluorescence >50 MESF, or…
   3. the CD61-APC fluorescence >17 MESF, or…
   4. the CD144-PE fluorescence >46 MESF.
5. skipping step 4, the diameter, as determined by Flow-SR, is between 200 nm and 650 nm, and…
6. the condition in Equation 2 is fulfilled, and…
   1. the refractive index, as determined by Flow-SR, is <1.42 to omit false positively labeled lipoproteins, and…
      1. the CD45-APC fluorescence >21 MESF, or…
      2. the CD62E-PE fluorescence >50 MESF, or…
      3. the CD61-APC fluorescence >17 MESF, or…
      4. the CD144-PE fluorescence >46 MESF.
   2. skipping step 6.1,
      1. the refractive index is <1.39, or…
      2. the refractive index is between 1.39 and 1.45, or…
      3. the refractive index is between 1.46 and 1.56.

| $\log_{10} I_{PE}>0.9x\cdot\log_{10} \sigma_{SSC}-1.0$ | Equation 3 |
| --- | --- |

Here, $I_{PE}$ is the fluorescence intensity of the PE detector and $\sigma_{SSC}$ is the effective side scattering cross section. PDF files with scatter plots of all applied gates are available via the online data repository (section 5.1). The fluorescence gates, which differentiate positively stained particles from background noise, were automatically determined with a publicly available MATLAB script using a tuning factor of 2.5 [7].

# References

[1] Théry C, Witwer KW, Aikawa E, Alcaraz MJ, Anderson JD, Andriantsitohaina R, Antoniou A, Arab T, Archer F, Atkin-Smith GK, others. Minimal information for studies of extracellular vesicles 2018 (MISEV2018): a position statement of the International Society for Extracellular Vesicles and update of the MISEV2014 guidelines. *J Extracell Vesicles* 2018; **7**: 1535750.

[2] Lee JA, Spidlen J, Boyce K, Cai J, Crosbie N, Dalphin M, Furlong J, Gasparetto M, Goldberg M, Goralczyk EM, others. MIFlowCyt: the minimum information about a Flow Cytometry Experiment. *Cytom Part A* Wiley Online Library; 2008; **73**: 926–30.

[3] Welsh JA, van der Pol E, Arkesteijn GJA, Bremer M, Brisson A, Coumans F, Dignat-George F, Duggan E, Ghiran I, Giebel B, Görgens A, Hendrix A, Lacroix R, Lannigan J, Libregts SFWM, Lozano-Andrés E, Morales-Kastresana A, Robert S, de Rond L, Tertel T, Tigges J, de Wever O, Yan X, Nieuwland R, Wauben MHM, Nolan JP, Jones JC. MIFlowCyt-EV: a framework for standardized reporting of extracellular vesicle flow cytometry experiments. *J Extracell Vesicles* 2020; **9**: 1713526.

[4] Buntsma NC, Shahsavari M, Gasecka A, Nieuwland R, van Leeuwen TG, van der Pol E. Preventing swarm detection in extracellular vesicle flow cytometry - a clinically applicable procedure. *Res Pract Thromb Haemost* Wiley; 2023; **7**: 100171.

[5] van der Pol E, de Rond L, Coumans FAW, Gool EL, Böing AN, Sturk A, Nieuwland R, van Leeuwen TG. Absolute sizing and label-free identification of extracellular vesicles by flow cytometry. *Nanomed Nanotechnol Biol Med* 2018; **14**: 801–10.

[6] de Rond L, Libregts SFWM, Rikkert LG, Hau CM, van der Pol E, Nieuwland R, van Leeuwen TG, Coumans FAW. Refractive index to evaluate staining specificity of extracellular vesicles by flow cytometry. *J Extracell Vesicles* 2019; **8**: 1643671.

[7] Gankema AAF, Li B, Nieuwland R, van der Pol E. Automated fluorescence gating and size determination reduce variation in measured concentration of extracellular vesicles by flow cytometry. *Cytom Part A* Wiley Online Library; 2022; **101**: 1049–56.

[8] Welsh JA, Arkesteijn GJA, Bremer M, Cimorelli M, Dignat-George F, Giebel B, Görgens A, Hendrix A, Kuiper M, Lacroix R, others. A compendium of single extracellular vesicle flow cytometry. *J Extracell Vesicles* Wiley Online Library; 2023; **12**: e12299.

1. EVs are modelled as core-shell particles with a core refractive index of 1.38, a shell refractive index of 1.48, and a shell thickness of 6 nm. [↑](#footnote-ref-1)
2. The effective scattering cross section is a hypothetical area of a particle that incoming light must impinge in order to be scattered towards the lens. The calibrated effective scattering cross section axis is independent of refractive index assumptions, but depends on the illumination wavelength and collection angles of the flow cytometer [8]. [↑](#footnote-ref-2)
3. The optical diameter equals the physical diameter of a particle when (1) the particle is spherical and (2) the particle has the same refractive index distribution as assumed in the model. [↑](#footnote-ref-3)
